# MicroRNA-455-5p Contributes to Cholangiocarcinoma Growth and Mediates Galangin's Anti-Tumor Effects

**DOI:** 10.7150/jca.58873

**Published:** 2021-06-04

**Authors:** Xu Deng, Meiling Zuo, Zhifang Pei, Yuanlin Xie, Zhongbao Yang, Zhihui Zhang, Minna Jiang, Dabin Kuang

**Affiliations:** 1 Department of Cardiology, The Third Xiangya Hospital, Central South University, Changsha, Hunan, China.; 2 Department of Pharmacy, The Affiliated Changsha Hospital of Hunan Normal University, Changsha, Hunan, China.; 3 Department of Cardiology, Xiangya Hospital, Central South University, Changsha, Hunan, China.

**Keywords:** cholangiocarcinoma, microRNA, galangin, survival, metastasis

## Abstract

Fully understanding the mechanism of how Cholangiocarcinoma (CCA) development and discovering promising therapeutic drugs are important to improve patients' survival time. This study identifies that microRNA-455-5p (miR-455-5p) targets protein phosphatase 1 regulatory subunit 12A (PPP1R12A), an effect that represses mitogen-activated protein kinase (MAPK) and PI3K/AKT pathway activation, thereby controlling CCA cells survival and metastasis. Moreover, miR-455-5p expression is reduced in CCA tissues and negative correlation with PPP1R12A and PPP1R12A knockdown phenotypic mimics miR-455-5p' effects on CCA cells. Furthermore, we demonstrate that galangin inhibits CCA growth both *in vitro* and *in vivo*, which is associated with increased miR-455-5p and repressed PPP1R12A expression. In support, overexpression of miR-455-5p abrogates those galangin-mediated anti-CCA effects. These findings establish an essential role of miR-455-5p in CCA development and galangin may provide a potential therapeutic adjuvant agent for anti-CCA treatment.

## Introduction

Cholangiocarcinoma (CCA) is a high malignant bile duct system tumor originating from the bile duct epithelial cells and accounts for about 3% of digestive system tumors. Evidence from clinic and experimental studies demonstrated that CCA is a very poor prognostic malignancy with a 5-year survive rate less than 5% due to its very early peripheral infiltration and distant metastasis 1-6. Traditional chemotherapy drugs, such as gemcitabine and platinum, have been showed to increase the median survival around 8-12 months 7-9. Yet, this still far from patient's anticipation and those chemotherapy drugs can also cause multiple serious side effects 10. Moreover, more than 70% of CCA patients are diagnosed in an advanced stage due to extraordinary invasiveness and not suit for surgical resection or liver transplantation 11-16. Therefore, an urgent needed in clinic to fully discover the mechanism behind CCA development and progression and promising targets for CCA treatment.

Accumulating evidence suggest that natural products derived from traditional Chinese herbal medicines are emerging as promising adjuvant drug to improve chemotherapy efficiency 17. For example, previous studies found that flavonoids had stronger anti-cancer effects with low toxicity which make them can serve as an ideal candidate for chemotherapeutic adjuvant 18. Galangin is a natural flavonoid product that extracted from the root of galangal and exhibits various anti-cancer effects against multiple tumor types (e.g. lung cancer, colon cancer and breast cancer) 19-22, rising a high possibility of galangin as an anti-cancer adjuvant agent. Although it has been reported that galangin suppressed CCA cells growth and metastasis 19, the underlying mechanism and whether it suitable serve as an adjuvant agent in anti-CCA treatment still largely unknown.

MicroRNAs (miRNAs) are a class of non-coding, single-stranded, small RNAs and play a crucial role in regulating gene expression at post-transcriptional level. It is well established that dysregulated miRNAs are important regulators involved in controlling various biological processes in tumor cells, such as proliferation, apoptosis, invasion, metastasis and others 23-27. Moreover, miRNAs not only contribute importantly to mediate multiple chemotherapy drugs' anti-cancer effects, but also involve in chemotherapeutic drug resistance 28-30. Herein, we find that miR-455-5p targets protein phosphatase 1 regulatory subunit 12A (PPP1R12A) and represses mitogen-activated protein kinase (MAPK) and PI3K/AKT pathway activation, thereby regulating CCA cells growth and metastasis. Moreover, using *in vitro* and tumor xenograft approaches, we show that miR-455-5p mediates galangin's anti-cancer effects on CCA. Our results establish an essential role of miR-455-5p in CCA development and galangin may provide a potential therapeutic adjuvant agent for anti-CCA treatment.

## Methods

### MiR-455-5p and protein phosphatase 1 regulatory subunit 12A expression data collection

We explored miR‐455-5p and protein phosphatase 1 regulatory subunit 12A (PPP1R12A) expression in CCA samples in TCGA Data Portal from ENCORI Pan-Cancer Analysis Platform (http://starbase.sysu.edu.cn/panCancer.php). Meanwhile, we also use this web server to analyze the correlation between miR-455-5p and PPP1R12A expression.

### CCA cell line culture and transfection

Two human CCA cell lines (TFK-1 and HCCC9810) and HEK 293T cells (CRL-3216) were obtained from the American Type Culture Collection (USA). TFK-1 and HCCC9810 cells were grown in RPMI-1640 medium (C11875500BT, Gibco, USA) supplemented with 10% fetal bovine serum (10270106, Gibco, USA) and antibiotics (100 U/ml penicillin and 0.1 mg/ml streptomycin) (P1400, Solarbio, China) in a humidified 5% CO_2_ incubator at 37°C. HEK 293T cells were cultured in DMEM supplemented 10% FBS and 1% penicillin and streptomycin. Cells passaged less than 8 times were used for all experiments. TFK-1 and HCCC9810 cells were plated at 5, 000 cells/well on 96-well plate, 120, 000 cells/well on 12-well plate or 250, 000 cells/well on 6-well plate, grown to 70%-80% confluency, and treated with galangin (282200, Sigma, USA) for 24 hours. For transfection experiments, cells were transfected with 100 nM of MiRNA non-specific mimic control (NS-m) (miR1N0000001-1-5, Ribobio, China), miR-455-5p mimic (455-m) (miR1N0003150-1-5, Ribobio, China), miRNA non-specific inhibitor (NS-i) (miR2N0000001-1-5, Ribobio, China), miR-455-5p inhibitor (455-i) (miR20003150-1-5, Ribobio, China), siRNA negative control (si-NC) (siN0000002-1-5, Ribobio, China) or PPP1R12A siRNA (si-PPP1R12A) (stB0004345B-1-5, Ribobio, China) and Lipofectamine 2000 transfection reagent from Invitrogen (11668019, USA) was used for transfection according to the manufacturer's instructions. After 14-16 hours of incubation, cells were replaced with fresh culture medium and incubated with another 12 hours before harvested for western blot and real-time PCR analysis. In some experiments, transfected cells were treated with galangin (150 μM) for 24 hours before used for further analysis.

### Cell proliferation assay

BeyoClick™ EdU Cell Proliferation Kit with Alexa Fluor 488 (C0071S, Beyotime, China) was used for cell proliferation analysis following the manufacturer's instructions. Briefly, TFK-1 and HCCC9810 cells were plated on 96-well plates at a density of 5, 000 cell/well and transfected miRNA mimic, miRNA inhibitor or siRNA with or without galangin (150 μM). Pre-warmed EDU-containing medium (20 μM) was added and incubated for 3 hours. Then cells were fixed with 4% paraformaldehyde for 15 min and followed by 0.3% triton-X 100 incubation at room temperature for 15 minutes. After 3 times wash with PBS, prepared Click System Solution was added into each well and incubated at 37 °C for 30 min in darkness. Then cells were washed with PBS for 3 times and incubated with Hoechst 33342 (0.5 μg/ml) at room temperature for 10 minutes. The numbers of Edu positive cells per view were observed under a fluorescence microscope and quantified from randomly acquired images.

### Cell apoptosis assay

TFK-1 and HCCC9810 cells were plated on 6-well plates at a density of 150, 000 cells/well and after indicated treatment. The apoptosis assay was performed using the Annexin V-FITC Apoptosis Detection Kit (C1062M, Beyotime, China) following the manufacturer's protocols. Cells were stained with Annexin V-FITC and propidium iodide (PI) in the dark. After washing with PBS, the apoptosis assay was examined by a flow cytometer (BD FACSCCanto II). Data were analyzed with FlowJo software (TreeStar, Inc. Ashland, OR).

### CCK-8 assay

TFK-1 and HCCC9810 cells were plated on 96-well plates at a density of 50, 000 cells/well and treated with different doses of galangin (12.5, 25, 50, 75, 100, 150, 200, 300, 400 μM) for 24 hours. After treatment, 10 μl CCK-8 (CK04, DOJIDO) was added into each well and incubated for 2 hours. The absorbance was measured at 450 nm using an ELISA plate reader (DTX880, Beckman).

### Cell migration and invasion assays

The 8 μm Transwell chamber (3422, Corning, USA) was used for the experiment. Before the start of the experiment, the diluted matrigel (354166, Corning, USA) (1:8) was used to spread in the upper chamber, and placed in the incubator for 2 hours (Migration experiment without this step). MiRNA mimic, miRNA inhibitor or si-RNA transfected TFK-1 and HCCC9810 cells with or without galangin treatment were adjusted to a density of 15, 000/200 μl using a serum-free medium and seeded in the upper chamber. 600μl of medium containing 10% FBS was added to the lower chamber. After 24 h, cells were fixed with 4% paraformaldehyde for 15 min and 0.1% crystal violet solution (G1062, Solarbio, China) for 15 min. Then wipe off the uninjured cells in the upper chamber and pictures were taken under a light microscope (Nikon, Japan).

### Dual-luciferase reporter assay

The wild-type (WT) and mutant-type (Mut) 3'-untranslated regions (UTRs) of PPP1R12A, which contain predicted binding sites for miR-455-5p, were synthesized and cloned to pmirGLO Dual-Luciferase miRNA Target expression vector (E1330, Promega, USA) by Vigene Biosciences (China). HEK 239T cells (150, 000 cells/well) were plated on 12-well plate, grown to 70% confluency, co-transfected with 250 ng WT or Mut luciferase reporter constructs, 10 ng Renilla plasmid (E2231, Promega, USA) and 100 nM 455-m or NS-m using lipofectamine 2000 according to the manufacturer's protocols. 24 hours after transfection, the cell lysates were collected and the luciferase activities were measured using the Dual-Glo Luciferase Assay System (E2920, Promega, USA) according to the manufacturer's instructions. Values were measured in a fluorescence reader (Veritas 9100-002, Turner Biosystems, USA). Each reading of luciferase activity was normalized to the Renilla activity.

### Real-time PCR

Tumor tissues were homogenized using TissueLyser II (Qiagen) according to manufacturer' instructions. Total RNA from treated TFK-1 and HCCC9810 cells or tumor tissues were isolated using Trizol reagent (9109, Takara, Japan) according to the manufacturer's instructions. 500 ng total RNA was reverse-transcribed to generate cDNA using the PrimeScript RT reagent kit (RR036A, Takara, Japan) according to the manufactures instruction. Real-time PCR analysis of PPP1R12A mRNA expression was performed using the TB Green Premix EX Tag kit (RR820A, Tarkara, Japan) with the Light Cycler 480 real-time PCR system (Rhoche) following the manufacturer's instruction. The primer sequences involved in the experiment were as follows: PPP1R12A*,* forward, GACAAAACCCCTGGCTTCTG; reverse, AGCTGCCCGTCTTTCTAAGT; GAPDH*,* forward, CTGCACCACCAACTGCTTAG, reverse, AGGTCCACCACTGACACGTT. Specific primers including miR-455-5p (MQPS0001464-1-100) and U6 (MQPS0000002-1-100) were purchased from RiboBio. The expression of targeted genes was analyzed using the 2^‑ΔΔCq^ method.

### Western blot analysis

Tumor tissues were homogenized using TissueLyser II (Qiagen) according to manufacturer' instructions. Protein from tissues and CCA Cells were isolated using RIPA buffer (P0013B, Beyotime, China) with phosphatase inhibitor (ST505, Beyotime, China). 20 μg of protein from each sample used for the 10% SDS-PAGE and then transferred to PVDF membrane (IPVH00010, Millipore, Germany). After transfer and blocking, membranes were incubated with following antibodies, Bax (1:1000, 60267, proteintech, China), Bcl-2 (1:1000, 60178, proteintech, China), caspase-3 (1:1000, 14220, CST, USA), Cleaved-Caspase3 (1:1000, 9664, CST, USA), MMP-9 (1:1000, 3852, CST, USA), Vimentin (1:1000, 3932, CST, USA), PPP1R12A (1:1000, ab32519, Abcam, UK), phospho-PI3K (1:1000, AP0854, Abclonal, China), phospho-Akt (1:1000, 4060, CST, USA), phospho-p38 (1:1000, 4511, CST, USA), phospho-JNK (1:1000, 9255, CST, USA), phospho-ERK1/2 (1:1000, 4370, CST, USA), and GAPDH (1:1000, ab8245, Abcam, UK). The membranes were washed using TBST and then incubated with correspondent secondary antibody. DAB Horseradish Peroxidase Color Development Kit was used for development (P0018S, Beyotime, China). The statistical gray value of each strip, and then calculate the relative expression.

### Subcutaneous xenograft animal model and Treatment

All animal experiments were approved by the Ethics Committee for Laboratory Animals of The Third Xiangya Hospital, Central South University, Hunan, China, and followed the Interdisciplinary Principles and Guidelines for the Use of Animals in Research, Testing, and Education by the New York Academy of Sciences, Ad Hoc Animal Research Committee. Four-weeks old Balb/c male nude mice were purchased from Hunan SJA Laboratory Animal Co. in China. TFK-1 cells (5 × 10^6^) were resuspended in 100 μl PBS and subcutaneously injected into the right flank of mice. When the tumor sizes were above 50 mm^3^, mice were randomly divided into two groups (*n* = 6 mouse per group) and intragastric treated with galangin (80 mg/kg/day) combined with intratumoral injected with lipofectamine 2000 encapsulated 455-i or NS-i (5 nM/mouse) according to protocol previously described in 31. The mice were sacrificed when they reach the experimental end point (day 28), and tumors were harvested for analysis.

### Immunohistochemistry

Tumor tissues were fixed with neutral buffered 10% formalin solution (HT501128, Sigma), embedded in paraffin, cut into sections at 4 μm, and deparaffinized. Antigen retrieval was performed using sodium citrate buffer (10 nM sodium and 0.05% Tween 20 at pH 6.0) at 96^o^C for 30 minutes. Sections were incubated with anti-Ki67 (1:50, A2094, Abclonal) or PPP1R12A (1:50, 22117010AP, proteintech, China) for 120 minutes at room temperature. Primary antibodies binding to tissue sections was visualized using DAB Detection kit (ZLI-9017, Zsbio, China), and counterstained with hematoxylin. All images were captured using a Microscope VS120 Whole Slide Scanner (Olympus) and analyzed using the computer-assisted Image-Pro Plus software (Meida Cybernetics, Bethesda, MD). The quantification of Ki67, and PPP1R12A staining was performed as positive area percent. Data were analyzed in a blinded fashion by two independent observers.

### Statistical analysis

All data are expressed as mean ± SEM. GraphPad 7.0 software package (GraphPad Software, Inc) was used for statistical analysis. Unpaired two-tailed student's *t* test was used to determine statistical significance between two groups normally distributed continuous variables. For multiple groups comparison, one-way ANOVA followed by Tukey multiple comparison analysis was used. For data without normal distribution, non-parametric Mann-Whitney *U* test or Kruskal-Wallis test was used. *P* < 0.05 was considered significant for all tests.

## Results

### Overexpression of miR-455-5p inhibits CCA cells proliferation and promotes apoptosis, migration and invasion

**Figure 1:**
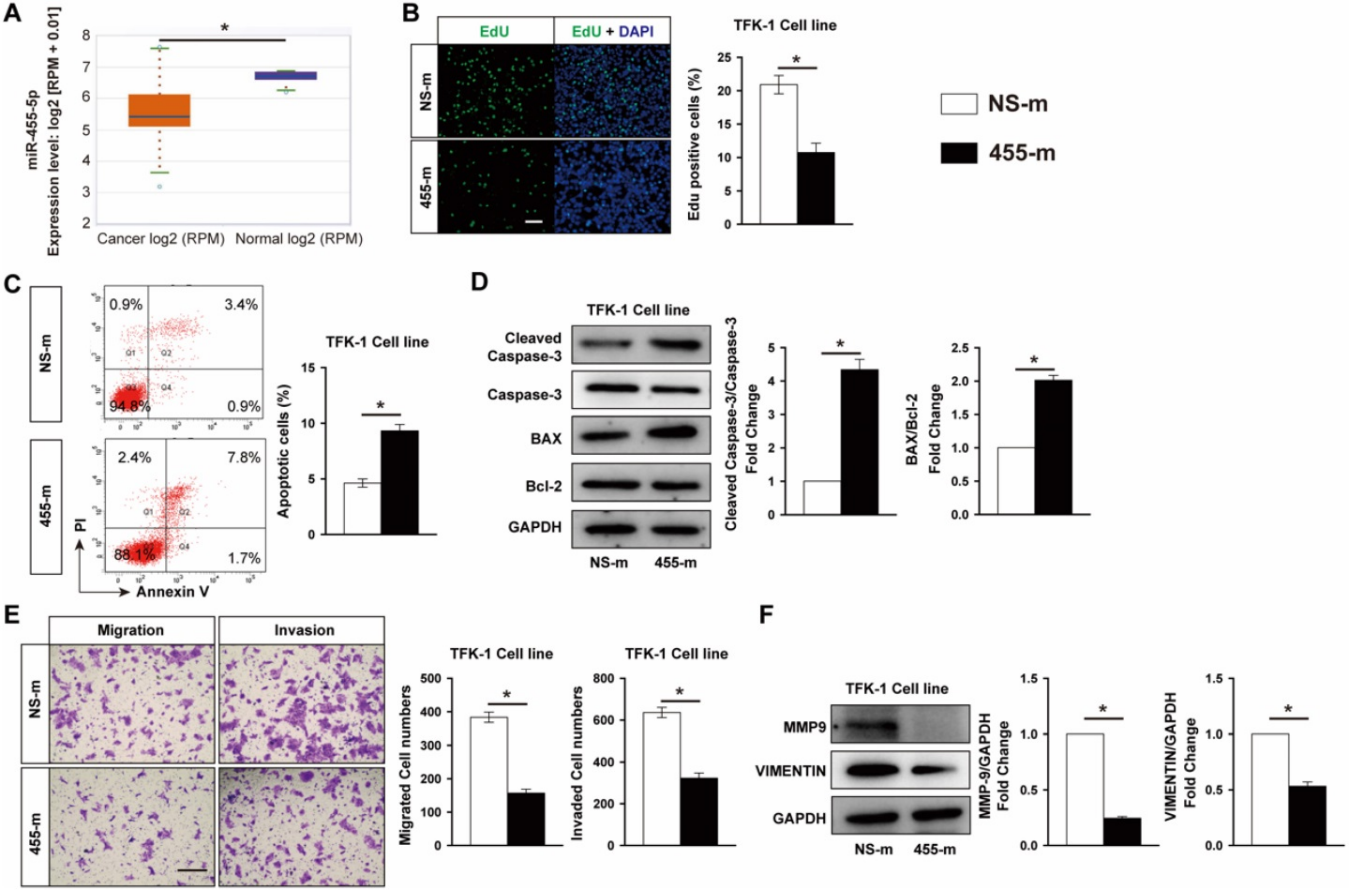
** MiR-455-5p is decreased in CCA cancer tissue samples and overexpression of miR-455-5p inhibits CCA cells proliferation, migration and invasion, but promotes apoptosis. (A)** CCA samples (n=36) and normal tissues (n=9) in TCGA dataset from ENCORI Pan-Cancer Analysis Platform showed that miR-455-5p expression was significantly down‐regulated in tumor tissues. TFK-1 cells were transfected with miR‐455-5p mimics (455-m) or non-specific mimic control (NS-m) at 100 nM for 24h. **(B)** EdU analysis of cell proliferation, Scale bar, 20 μM. **(C)** FACS analysis of cell apoptosis. **(D)** Western blot analysis of cleaved caspase 3, Caspase 3, Bax and Bcl-2 protein expression. **(E)** Matrigel-coated Transwell analysis of migration and invasion, scale bar, 50 μm. **(F)** Western blot analysis of MMP9 and Vimentin protein expression. **B to F**, *n* = 3 independent experiments. Values were given as means ± SEM. *P < 0.05.

MiR-455-5p is an high conserved miRNA and have been shown to contribute importantly to multiple tumors development 25, 27, 32-35. To investigate the role of miR-455-5p in CCA, we first performed an integrative analysis of CCA samples from The Cancer Genome Atlas (TCGA) database using ENCORI Pan-Cancer Analysis Platform. A total of 45 samples, including 9 normal and 36 CAA tissues, were extracted from database and the analysis revealed that the expression of miR-455-5p was significantly reduced in CAA tissues compared with normal tissue (**Fig. 1A**), indicating that miR-455-5p may contribute to CCA development. To assess the potential role of miR-455-5p in CCA development, we examined the effect of miR-455-5p on cell survival and metastasis in CCA cells by using gain-function experiments. As shown in **Fig. 1B and Fig. S1A**, compared with non-specific mimic control (NS-m) transfected CCA cell line, including bile duct carcinoma TFK-1 cells and intrahepatic bile duct carcinoma HCCC9810 cells, EdU staining assay exhibited that overexpression of miR-455-5p using miR-455-5p mimic (455-m) inhibited TFK-1 and HCCC9810 cells proliferation by 48.6% and 52.2%, respectively. We next investigated the effect of miR-455-5p on CCA cells apoptosis by flow cytometry (FACS) and western blot. In CCA cell lines transfected with miR-455-5p mimic, we observed a 2.02-fold induction of apoptotic cells in TFK-1 cells and 2.46-fold increase in HCCC9810 cells compared with cells transfected with NS-m (**Fig. 1C and Fig. S1B**). In agreement with this observation, overexpression of miR-455-5p increased the ratio of Bax to Bcl-2 and expression of cleaved caspase-3 at protein levels in both TFK-1 and HCCC9810 cell line (**Fig. 1D and Fig. S1C**). To further determine whether miR-455-5p also involved in metastasis, we performed transwell analysis to assess the effect of miR-455-5p on CCA cell migration and invasion. As shown in **Fig. 1E**, overexpression of miR-455-5p reduced TFK-1 cells migration by 59.1% and invasion by 49.2%. Similar phenotypic change was also observed in miR-455-5p transfected HCCC9810 cells (**Fig. S1D**). It is well established that epithelial-to-mesenchymal transition (EMT) and matrix metalloproteinases (MMPs; e.g. MMP9) play crucial roles in mediating tumor migration and invasion 36. VIMENTIN is an important mesenchymal marker, we found that overexpression of miR-455-5p significantly reduced MMP-9 and VIMENTIN expression at protein levels in both HCCC9810 cells (**Fig. 1F**) and TFK-1 cells **(Fig. S1E)**. The induction of miR-455-5p by miR-455-5p mimic transfection in both HCCC9810 and TFK-1 cells was confirmed by real-time PCR analysis (**Fig. S2**). Collectively, these data suggested that miR-455-5p is able to negatively affect CCA cells survival, apoptosis, migration and invasion.

### PPP1R12A is a functional target of miR-455-5p and inhibition of PPP1R12A expression suppresses CCA cells survival and metastasis

**Figure 2:**
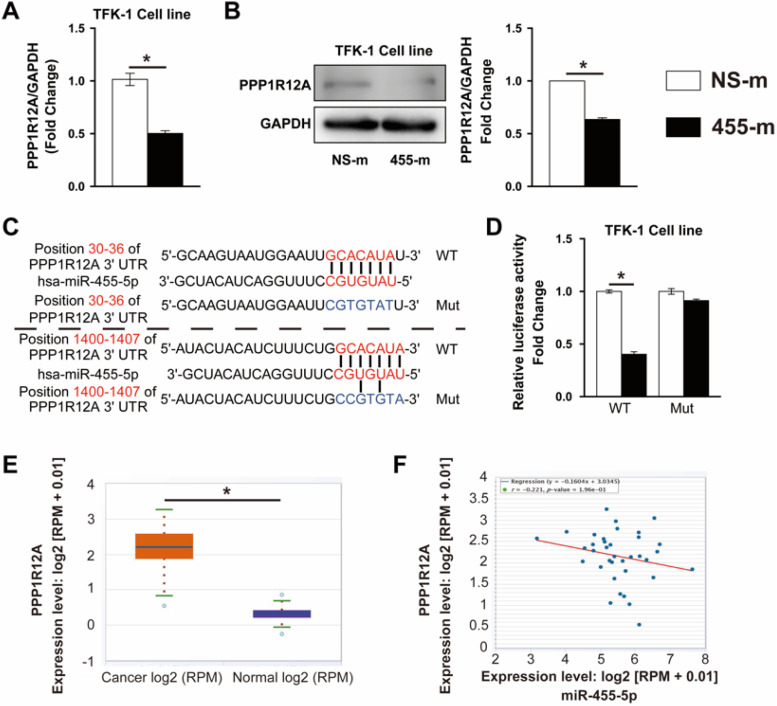
** PPP1R12A is a direct target of miR-455-5p and negatively correlated with miR-455-5p expression in CCA tissue samples.** TFK-1 cells were transfected with miR‐455-5p mimic (455-m) or non-specific mimic control (NS-m) at 100 nM for 24h.** (A)** Real-time PCR analysis of PPP1R12A mRNA expression, *n* = 3 independent experiments. **(B)** Western blot analysis of PPP1R12A protein expression, *n* = 3 independent experiments. **(C)** Bioinformatics analysis indicated potential 3'UTR position of miR‐455‐5p on PPP1R12A. **(D)** Luciferase reporter assay of PPP1R12A 3'-UTR activity in HEK 293T cells,* n* = 3 independent experiments. **(E)** CCA samples (n=36) and normal tissues (n=9) in TCGA dataset from ENCORI Pan-Cancer Analysis Platform showed that PPP1R12A expression was significantly up‐regulated in tumor tissues. **(F)** The expression of miR‐455-5p in CCA tissues was negatively correlated with the level of PPP1R12A mRNA. Values are given as means ± SEM. *P < 0.05.

It is well established that miRNAs exert its function through regulating targeted gene expression at post-transcriptional level. To identify the potential target regulated by miR-455-5p, we performed bioinformatics assay using TargetScan 37 and miRWalk 38 algorithms and PPP1R12A, one of myosin phosphatase subunits controlling cell cycle and migration 25, 34, 39, was predicted to be a miR-455-5p target. In miR-455-5p-overexpressed TFK-1 cells, PPP1R12A expression was reduced by 51% at mRNA level and 40% at protein level (**Fig. 2A and 2B**). TargetScan algorithm indicated that 2 potential miR-455-5p-binding sites in the 3' UTR of PPP1R12A (**Fig. 2C**). Overexpression of miR-455-5p inhibited the activity of a luciferase reporter construct containing both PPP1R12A 3'UTR, while not the PPP1R12A construct with mutation sites at predicated site at 3'UTR region (**Fig. 2D**). Moreover, PPP1R12A expression and correlation analysis using the CCA samples from TCGA database exhibited that the expression of PPP1R12A was significantly increased in CCA cancer samples (**Fig. 2E**) and a negative correlation between miR-455-5p and PPP1R12A in CCA samples (**Fig. 2F**). Taken together, these data suggested that PPP1R12A may play an importantly role in CCA and miR-455-5p-mediated CCA cell survival and metastasis may through modulating PPP1R12A expression.

**Figure 3:**
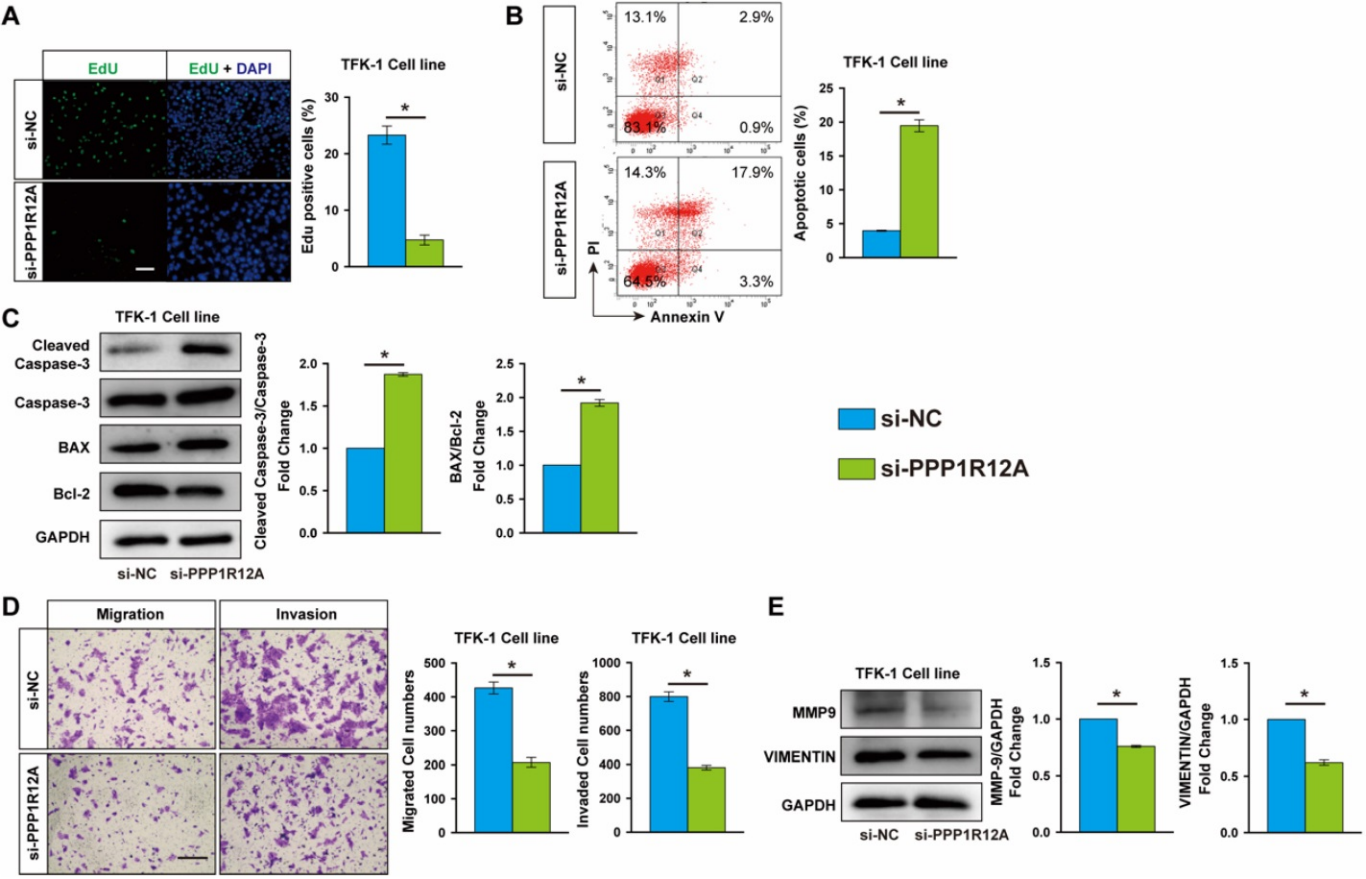
** PPP1R12A knockdown suppresses CCA cells proliferation and promotes apoptosis.** TFK-1 cells were transfected with siRNA negative control (si-NC) or PPP1R12A siRNA (si-PPP1R12A) at 100 nM for 24h. **(A)** EdU analysis of cell proliferation, Scale bar, 20 μM. **(B)** FACS analysis of cell apoptosis. **(C)** Western-blot analysis of cleaved caspase 3, Caspase 3, Bax and Bcl-2 expression at protein level. **(D)** Matrigel-coated Transwell analysis of migration and invasion, scale bar, 50 μm. **(E)** Western blot analysis of MMP9 and Vimentin protein expression. **A to E**, *n* = 3 independent experiments. Values are given as means ± SEM. *P < 0.05.

To assess the potential role of PPP1R12A in CCA, we examined the effect of PPP1R12A on CCA cells survival and metastasis by using loss-of-function experiments. EdU assay exhibited that Inhibition of PPP1R12A expression dramatically decreased TFK-1 and HCCC9810 cells proliferation by 79.7% and 72.3%, respectively (**Fig. 3A and Fig. S3A**). Moreover, cell apoptosis measurement by FACS using Annexin V and PI double staining showed that inhibition of PPP1R12A expression promoted both TFK-1 and HCCC9810 cells apoptosis by 4.9-fold and 2.33-fold, respectively (**Fig. 3B and Fig. S3B**). Consistent with these results, in TFK-1 and HCCC9810 cells transfected with PPP1R12A siRNA exhibited a significantly increased ratio of Bax to Bcl-2 and cleaved caspase-3 expression at protein levels compared with siRNA negative control transfected cells (**Fig. 3C and Fig. S3C**). Furthermore, Transwell assay showed that silencing PPP1R12A significantly decreased the numbers of migrated and invaded cells compared with siRNA negative control transfected HCCC9810 and TFK-1 cells (**Fig. 3D and Fig. S3D**). Consistently, the expression of MMP9 and VINMENTIN at protein levels in both PPP1R12A siRNA-transfected HCCC9810 and TFK-1 cells were significantly decreased compared with those cells transfected with siRNA negative control (**Fig. 3E and Fig. S3E**). The efficiency of PPP1R12A siRNA in TFK-1 and HCCC9810 cells was confirmed by Real-time PCR and western blot (**Fig. S4A and S4B**). In summary, these data (**Fig. 2, Fig. 3 and Fig. S3**) demonstrated that inhibition of PPP1R12A expression suppresses CCA cells survival and metastasis and may mediate the anti-tumor effects of miR-455-5p.

### MiR-455-5p expression is induced by galangin treatment and mediates the anti-tumor effects of galangin on CCA

**Figure 4:**
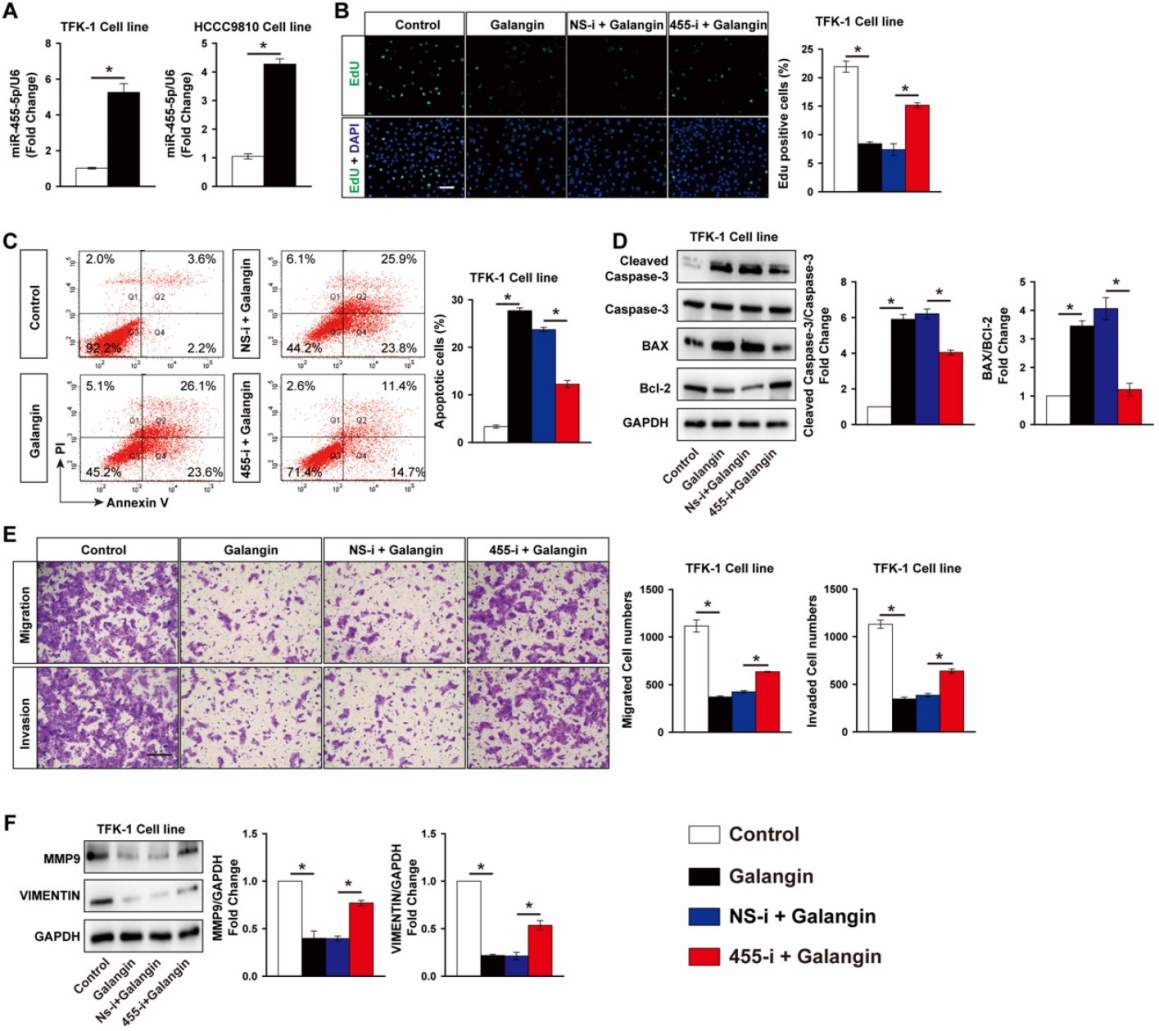
** Inhibition of miR-455-5p abrogates galangin's anti-cancer effects on CCA cells. (A)** Real-time PCR analysis of miR-455-5p expression in TFK-1 and HCCC9810 cells treated with galangin at 150 μM for 24h. TFK-1 cells were transfected with miR‐455-5p inhibitor (455-i) or non-specific inhibitor control (NS-i) at 100 nM for 24h followed by 150 μM galangin treatment for another 24h and harvested for **(B)** Edu analysis, Scale bar, 20 μM. **(C)** FACS analysis of apoptosis. **(D)** Western blot analysis of cleaved caspase 3, Caspase 3, Bax and Bcl-2 protein expression. **(E)** Matrigel-coated Transwell analysis of cell migration and invasion, scale bar, 50 μm. **(F)** Western blot analysis of MMP9 and Vimentin protein expression. **A to F**, *n* = 3 independent experiments. Values are given as means ± SEM. *P < 0.05.

Galangin is a natural flavonoid product extracted from the root of galangal and exhibits multiple anticancer effects against various tumors without serious side effects. We found that galangin treatment significantly decreased TFK-1 cells viability in a dose-dependent manner (**Fig. S5**). Unexpectedly, galangin potently increased miR-455-5p expression by 5.3-fold in TKF-1 cells and 4.2-fold in HCCC9810 cells, respectively (**Fig. 4A**), suggesting miR-455-5p may mediate the anti-tumor effects of galangin on CCA cells. To test this hypothesis, miR-455-5p antagomir was used to inhibit miR-455-5p expression in both TFK-1 and HCCC9810 cells (**Fig. S6**). Edu assay demonstrated that galangin treatment significantly decreased TFK-1 Cell proliferation, but not in TFK-1 cells transfected with miR-455-5p inhibitor (**Fig. 4B**). Moreover, FACS showed that galangin treatment increased TKF-1 and HCCC9810 cells apoptosis by 8.3- and 6.8-fold, respectively (**Fig. 4C, Fig. S7A**). These phenotypic alterstions were further confirmed by western blot assay using apoptotic marker cleaved caspase-3, Bcl2 and Bax (**Fig. 4D, Fig. S7B**). Furthermore, in CCA cells treated with galanin exhibited less migrated and invaded cells (**Fig. 4E, Fig. S7C**). In agreement with these results, the protein level of MMP9 and Vimentin were lower in both TKF-1 and HCCC9810 cells treated with galanin (**Fig. 4F, Fig. S7D**). In contrast, these galanin-mediated anti-tumor effects on CCA cells were abrogated by inhibition of miR-455-5p expression (**Fig. 4C to 4F, Fig. S6 and S7**).

**Figure 5:**
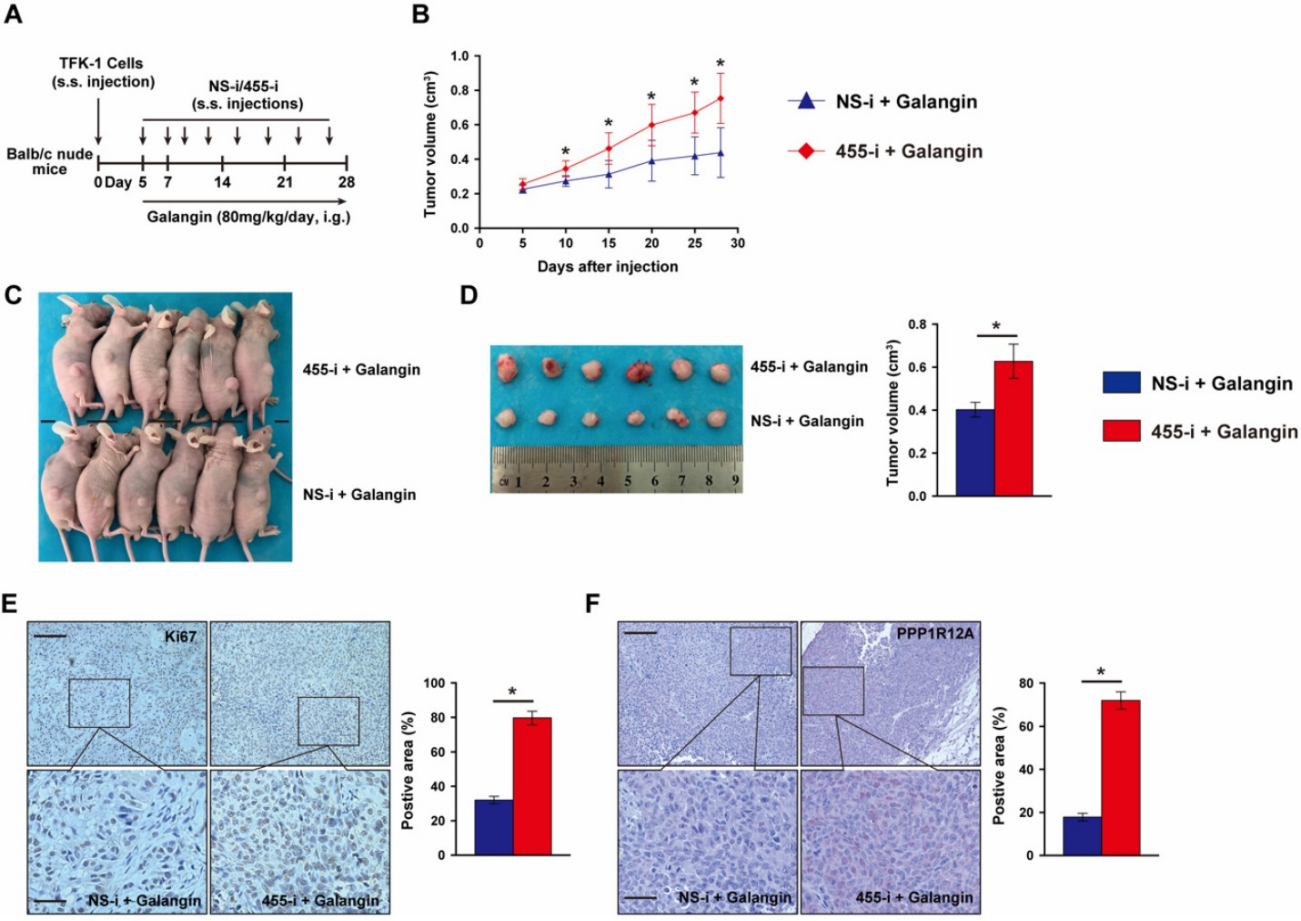
** MiR-455-5p knockdown abrogates Galangin-suppressed xenograft expansion *in vivo*. (A)** Schematic illustration for the xenograftr modeling and galanign with non-specific inhibitor (NS-i) or miR-455-5p inhibitor (455-i) treatment. **(B)** The tumor growth curve over time of Balb/c nude mice mice treatment with galangin and NS-i or 455-i, respectively. **(C)** Tumor image of xenograft tissues after mice received galangin and NS-i or 455-i at day 28, respectively. **(D)** Tumor imaging and quantification of tumor volume. **(E)** Immunohistochemistry staining of Ki67 and quantification in xenograft tissues after mice received galangin and NS-i or 455-i, respectively. Scar bar 100 μM, insert 50 μM. **(F)** Immunohistochemistry staining of PPP1R12A expression and quantification in xenograft tissues after mice received galangin and NS-i or 455-i, respectively. Scar bar 100 μM, insert 25 μM. *n* = 6 mice per group. Values are given as means ± SEM. *P< 0.05.

To further evaluate whether the anti-tumor effects of galangin on CCA depends on miR-455-5p expression and galangin could use for CCA therapeutics, we performed a tumor xenograft by subcutaneously injecting TFK-1 cells with miR-455-5p knockdown and galangin treatment (**Fig. 5A**). Compare to galangin treated Balb/c nude mice with NS-i injection, inhibition of miR-455-5p expression in mice received galangin treatment exhibited an expanded growth of TKF-1 cell xenograft (**Fig. 5B to 5D, Fig. S8**). Moreover, an increased proliferation marker Ki67 was observed in galangin treated mice with miR-455-5p inhibitor (**Fig. 5E**). Furthermore, PPP1R12A expression determined by immunohistochemistry staining was significant higher in galangin and 455-i treated Balb/c nude mice than those mice treatment with galangin and NS-i (**Fig. 5F**). Taken together, these findings suggest that the therapeutic effects of galangin on CCA and that miR-455-5p mediates the anti-tumor effects of galangin.

### MiR-455-5p expression contributes importantly to galangin-mediated MAPK and PI3K/AKT pathway activity in CCA cells

**Figure 6:**
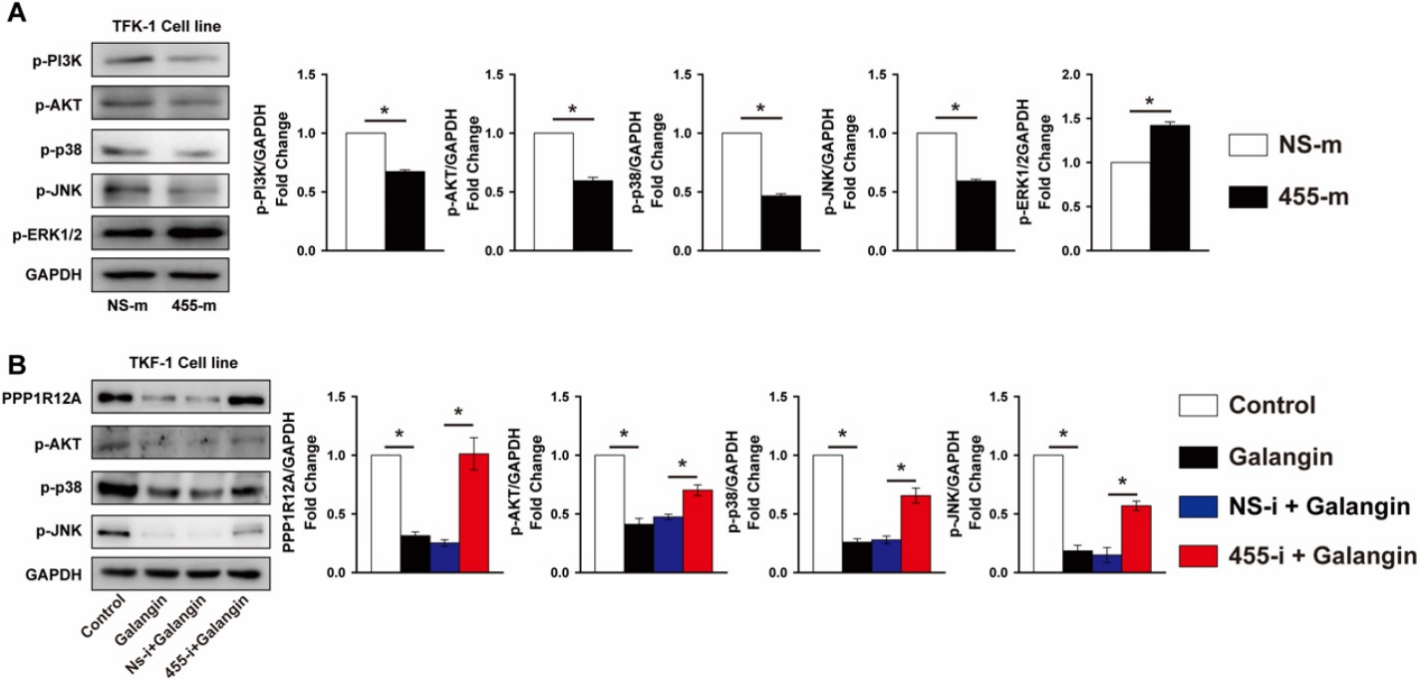
** miR-455-5p regulates PI3K/AKT and MAPK signal pathway activity and mediates galangin's effects***.*** (A)** Western blot analysis of phospho-PI3K, phopspho-AKT, phospho-p38, phospho-JNK and phospho-ERK1/2 expression at protein level in TFK-1 cells transfected with miR-455-5p mimic (455-m) or non-specific mimic control (NS-m) for 24h. **(B)** Western blot analysis of PPP1R12A, phospho-AKT, phospho-p38, phospho-JNK expression at protein level in TFK-1 cells transfected with miR-455-5p inhibitor (455-i) or non-specific inhibitor control (NS-i) for 24h, followed by galangin (150 μM) treatment for 24h. **A and B**, *n* = 3. Values are given as means ± SEM. *P < 0.05.

Previous studies demonstrated that the MAPK and PI3K/Akt signal pathway contributed greatly to tumor cells survival and metastasis 40, 41. As shown in **Fig. 6A**, overexpression of miR-455-5p reduced the phosphorylation of p38 and JNK by 53% and 40% in TFK-1 cells, respectively, but increased ERK1/2 phosphorylation by 42% (**Fig. 6A**). Analogously, the phosphorylation of PI3K and AKT were also decreased by 33% and 40%, respectively, in TFK-1 cells transfected with miR-455-5p mimic compared to NS-m transfected cells (**Fig. 6A**). Similarly, galangin treatment significantly decreased phosphorylation of p38, JNK and AKT, as well as PPP1R12A expression (**Fig. 6B**). In contrast, inhibition of miR-455-5p expression abrogated these galangin-mediated phenotypic changes in TFK-1 cells (**Fig. 6B**). Collectively, these data indicate that miR-455-5p affects CCA cells survival and metastasis and mediates galangin's anti-tumor effects may through modulating PPP1R12A expression and MAPK and PI3K/AKT pathway.

## Disscusion

In the present study, we provide evidence that the miR-455-5p expression was reduced in human CCA tissues and play critical roles in controlling CCA cells survival and metastasis. Moreover, we identified that PPP1R12A was a directly target of miR-455-5p and negative correlated with miR-455-5p expression in human CCA tissues, and PPP1R12A knockdown mimics miR-455-5p' effects on CCA cells. Furthermore, we found that galangin inhibited CCA growth both *in vitro* and *in vivo*, which was associated with increased miR-455-5p and decreased PPP1R12A expression. In support, using a loss-of-function approach, we demonstrated that inhibition of miR-455-5p expression abrogated galangin's anti-tumor effects. Finally, miR-455-5p overexpression and galangin treatment inhibited MAPK and PI3K/AKT pathway activity. Taken together, these data indicate that miR-455-5p play a critical role in controlling CCA growth and mediating the anti-tumor effects of galangin, at least in part, through targeting PPP1R12A and modulating MAPK and PI3K/AKT pathway.

Accumulating evidence demonstrated that miRNAs play an important role in tumor growth and metastasis by regulating protein expression at the post-transcriptional level 42. For instance, miR-455-5p is an high conserved miRNA and play critical roles in multiple tumors development and progression 25, 27, 32-35, 39, 43. Yet, the expression profile and role of miR-455-5p in CCA development and progression remains largely unknown. In the current study, we showed that the expression of miR-455-5p was reduced in CCA tissues compared to adjacent normal tissues. Previously studies identified that miRNAs enriched in a tissue- or cell-specific manner that can exact different biological functions by modulating large gene networks expression 44, 45. This may explain why the expression of miR-455-5p was different to oral carcinoma and colon cancer. Findings from other groups suggested that miR-455-5p affected tumor growth largely dependent on modulating cell proliferation and apoptosis, an effect promoting tumor migration and invasion 39, 46, 47. For example, overexpression of miR-455-5p suppressed prostate cancer cell proliferation and inhibited tumor growth by targeting C-C motif chemokine receptor 5 47. In colon cancer, miR-455-5p targeted galectin-9 expression, resulting in increased HT29 cells apoptosis and reduced proliferation 39. In concordance with these observations, we demonstrated that miR-455-5p overexpression potently inhibited CCA cells proliferation, migration and invasion, whereas promoted apoptosis by using multiple complementary methods. Moreover, we demonstrated that PPP1R12A, a subunit of myosin phosphatase that play an critical role in regulating cell cycle and migration 25, 34, 39, was a new direct target of miR-455-5p and PPP1R12A expression was negatively correlated with miR-455-5p in CCA tissues. Indeed, previous studies found that PPP1R12A interacted with insulin receptor substrate 1-insulin-like growth factor 1 receptor (IRS1-IGF1R) complex and mediated the process of IRS1 dephosphorylation, promoting PI3K/AKT cascade activation and in turn led to tumor cell proliferation and tumor growth 43, 48. In gastric cancer (GC) cells, silencing PPP1R12A inhibited GC cells proliferation by promoting cyclin D1 and c-myc expression to block the cell cycle 49. Our findings also found that silencing PPP1R12A exhibited stronger anti-turmor effects on CCA cells that consistent with miR-455-5p overexpression. Taken together, these data suggest that miR-455-5p may use as a potential therapeutic target of CCA and the effects of miR-455-5p on suppressing CCA cells survival and metastasis, at least in part, through targeting PPP1R12A.

Accumulating evidence suggests that multiple signal pathways are playing critical roles in mediating cell proliferation, migration and invasion, the hallmarks of tumor growth and metastasis 50, 51. Among those pathways, PI3K/AKT and MAPK pathways are probably the best known and interesting targets involved in preclinical and clinical trials for cancer treatment 52. In the current study, we found that miR-455-5p overexpression significantly decreased PI3K/AKT phosphorylation in both HCCC9810 and TFK-1 cells, as well as p38 and JNK, two major MAPK downstream kinases, resulting in a pro-apoptotic phenotype. Together, these findings indicate that miR-455-5p inhibits MAPK and PI3K/AKT pathway activation, which in turn modulating CCA cells growth and metastasis.

Although results from experimental and clinic studies demonstrate that gemcitabine and platinum is the first-line treatment of CCA patients 53, 54, the side effects and drug resistance remarkable limit those chemotherapy agents using in patients. Moreover, the median survival time in CCA patients by using those drugs is less than 8 to 12 months 7-9, making urgent clinical needed to develop novel therapeutic agents or discover appropriate adjuvant for CCA treatment. Accumulating studies identified that natural products, such as flavonoids, might be an eligible choice due to its stronger anti-tumor effects and low toxicity 18. Galangin is a flavonoid derived from the root of galangal and exhibits various anti-tumor effects against multiple cancer types 20-22, 55, 56. For instance, galangin inhibited glioma cells proliferation, metastasis, and angiogenesis by suppressing CD44 expression, a hall marker in glioma and associated with EMT 20. Moreover, in retinoblastoma, galangin potently repressed cell progression and induced cell apoptosis through activating PTEN and caspase-3 pathways 55. Furthermore, galangin induced gastric cancer cells apoptosis via regulation of ubiquitin carboxy-terminal hydrolase isozyme L1 and glutathione S-transferase P 56. In agreement with a recently published report 19, we found that galangin treatment potently induced CCA cells apoptosis and repressed proliferation and metastasis. Moreover, we added new evidence that, using both *in vitro* and *in vivo* approaches, the galangin inhibits CCA growth depended on miR-455-5p expression. Indeed, accumulating evidence suggested that galangin exhibits its anti-tumor effect not only act as a switch to regulate a specific, individual gene expression but rather to modulate expression of large gene networks (e.g. miR-21, H19, p53, CD44, and so on) 19, 20, 57, 58. Collectively, these results suggest that galangin represents a high possibility as an adjuvant to treat CCA.

In summary, our findings demonstrate that miR-455-5p mediates galangin's anti-tumor effect and inhibits CCA cells proliferation, migration, and invasion, at least in part, by targeting PPP1R12A, an effect that decreases MAPK and PI3K/Akt pathway activation in CCA cells. Therefore, strategies targeting miR-455-5p can be serve as a potential therapeutic target for CCA and galangin may provide as novel treatment choice to improve chemotherapy efficiency.

## Supplementary Material

Supplementary figures and tables.
