# Supplementary material for: MicroRNA-455-5p Contributes to Cholangiocarcinoma Growth and Mediates Galangin's Anti-Tumor Effects
Source: J Cancer. 2021 Jun 4;12(15):4710–21. doi: 10.7150/jca.58873 (PMC8210562; doi:10.7150/jca.58873)
Supplement: Supplementary file 1 — Supplementary figures and tables. [file jcav12p4710s1.pdf]

MicroRNA-455-5p Contributes to Cholangiocarcinoma Growth and Mediates Galangin's Anti-Tumor effects

Authors: Xu Deng[1], Meiling Zuo[2], Zhifang Pei[3], Yuanlin Xie[2], Zhongbao Yang[2], Zhihui Zhang[1], Minna Jiang[3*], Dabin Kuang[2*]

Affiliations:

[1]Department of Cardiology, The third Xiangya Hospital, Central South University, Changsha, Hunan, China

[2]Department of Pharmacy, The Affiliated Changsha Hospital of Hunan Normal University, Changsha, Hunan, China

[3]Department of Cardiology, Xiangya Hospital, Central South University, Changsha, Hunan, China

Supplemental Figure 1. Overexpression of miR-455-5p inhibits CCA cells proliferation, migration and invasion, but promotes apoptosis.

Supplemental Figure 2. The efficiency of miR-455-5p mimics transfection in TFK-1 and HCCC9810 cells.

Supplemental Figure 3. Inhibition of PPP1R12A represses proliferation, migration and invasion, but promotes apoptosis in HCCC9810 cells.

Supplemental Figure 4. The efficiency of PPP1R12A knockdown in TFK-1 and HCCC9810 cells.

Supplemental Figure 5. Galangin inhibits TFK-1 cells cell viability in a dose-dependent manner.

Supplemental Figure 6. The efficiency of miR-455-5p inhibitor transfection in TFK-1 and HCCC9810 cells.

Supplemental Figure 7. Inhibition of miR-455-5p abrogates galangin's anti-cancer effects on CCA cell line HCCC9810 cells.

Supplemental Figure 8. The efficiency of miR-455-5p knockdown in xenograft model.

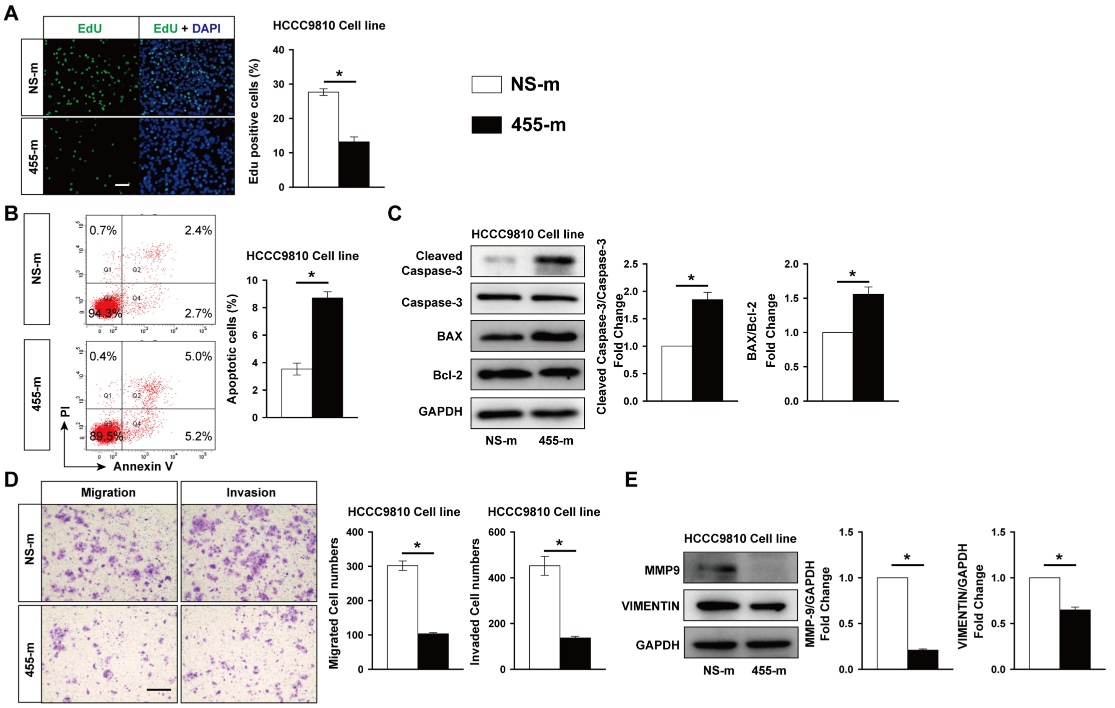

Supplemental Figure 1. Overexpression of miR-455-5p inhibits CCA cells proliferation, migration and invasion, but promotes apoptosis. HCCC9810 cells were transfected with miR-455-5p mimics (455-m) or non-specific mimic control (NS-m) at 100 nM for 24h. **(A)** EdU analysis of cell proliferation, Scale bar, 20 μM. (**B**) FACS analysis of cell apoptosis. (**C**) Western blot analysis of Bax, Bcl-2, cleaved caspase 3 and Caspase 3 protein expression. (**D**) Matrigel-coated Transwell analysis of migration and invasion, scale bar, 50 μm. **(E)** Western blot analysis of MMP9 and Vimentin protein expression. **A to E**, *n* = 3 independent experiments. Values were given as means ± SEM. *$P < 0.05$.

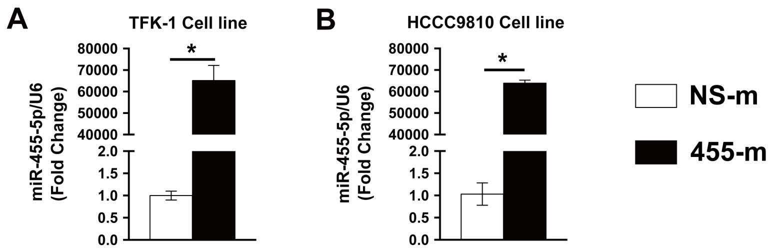

Supplemental Figure 2. The efficiency of miR-455-5p mimics transfection in TFK-1 and HCCC9810 cells. CCA cells were transfected with miR-455-5p mimics (455-m) or non-specific mimic control (NS-m) at 100 nM for 24h. **(A)** Real-time PCR analysis of miR-455-5p expression in 455-m or NS-m transfected TFK-1 cells. **(B)** Real-time PCR analysis of miR-455-5p expression in 455-m or NS-m transfected HCCC9810 cells. **A and B**, n = 3 independent experiments. Values were given as means ± SEM. $^*P < 0.05$.

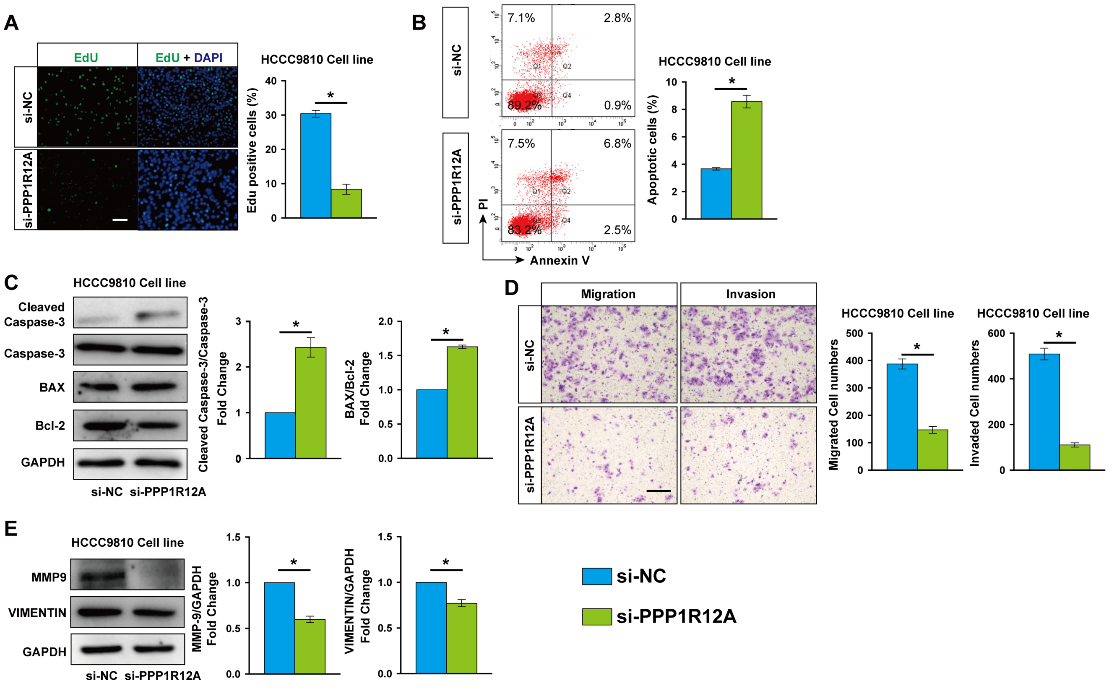

Supplemental Figure 3. Inhibition of PPP1R12A represses proliferation, migration and invasion, but promotes apoptosis in HCCC9810 cells.

HCCC9810 cells were transfected with siRNA negative control (si-NC) or PPP1R12A siRNA (si-PPP1R12A) at 100 nM for 24h. (**A**) EdU analysis of cell proliferation, Scale bar, 20 μM. (**B**) FACS analysis of cell apoptosis. (**C**) Western-blot analysis of Bax, Bcl-2, cleaved caspase 3 and Caspase 3 expression at protein level. (**D**) Matrigel-coated Transwell analysis of migration and invasion, scale bar, 50 μm. (**E**) Western blot analysis of MMP9 and Vimentin protein expression. **A to E**, *n* = 3 independent experiments. Values are given as means ± SEM. *$P < 0.05$.

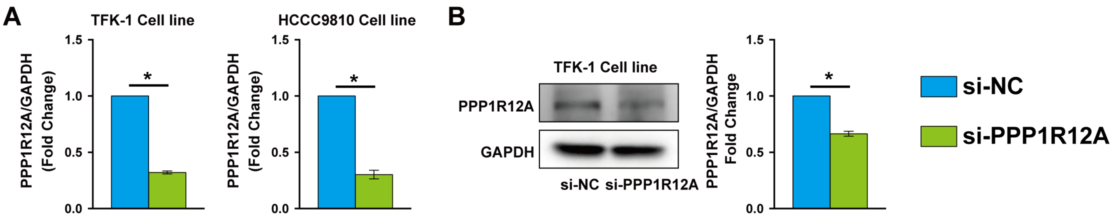

Supplemental Figure 4. The efficiency of PPP1R12A knockdown in TFK-1 and HCCC9810 cells. CCA cells were transfected with siRNA negative control (si-NC) or PPP1R12A siRNA (si-PPP1R12A) at 100 nM for 24h. **(A)** Real-time PCR analysis of PPP1R12A mRNA expression in si-NC or si-PPP1R12A transfected TFK-1 and HCCC9810 cells. **(B)** Western blot analysis of PPP1R12A protein expression in 4 si-NC or si-PPP1R12A transfected TFK-1 cells. **A and B**, *n* = 3 independent experiments. Values were given as means ± SEM. *$P < 0.05$.

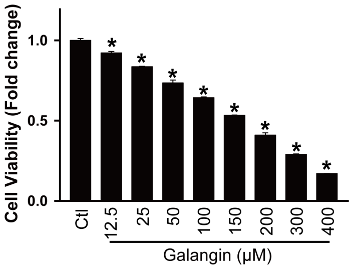

Supplemental Figure 5. Galangin inhibits TFK-1 cells cell viability in a dose-dependent manner. TFK-1 cells were treated with different doses of galangin for 24 hours and harvested for CCK-8 analysis. *n* = 3 independent experiments. Values were given as means ± SEM. *$P < 0.05$.

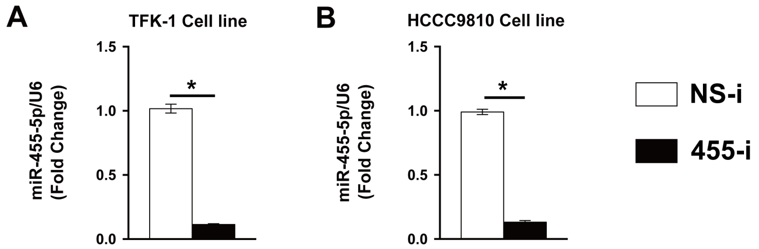

Supplemental Figure 6. The efficiency of miR-455-5p inhibitor transfection in TFK-1 and HCCC9810 cells. CCA cells were transfected with miR-455-5p inhibitor (455-i) or non-specific inhibitor control (NS-i) at 100 nM for 24h. **(A)** Real-time PCR analysis of miR-455-5p expression in 455-i or NS-i transfected TFK-1 cells. **(B)** Real-time PCR analysis of miR-455-5p expression in 455-i or NS-i transfected HCCC9810 cells. **A and B**, n = 3 independent experiments. Values were given as means ± SEM. *$P < 0.05$.

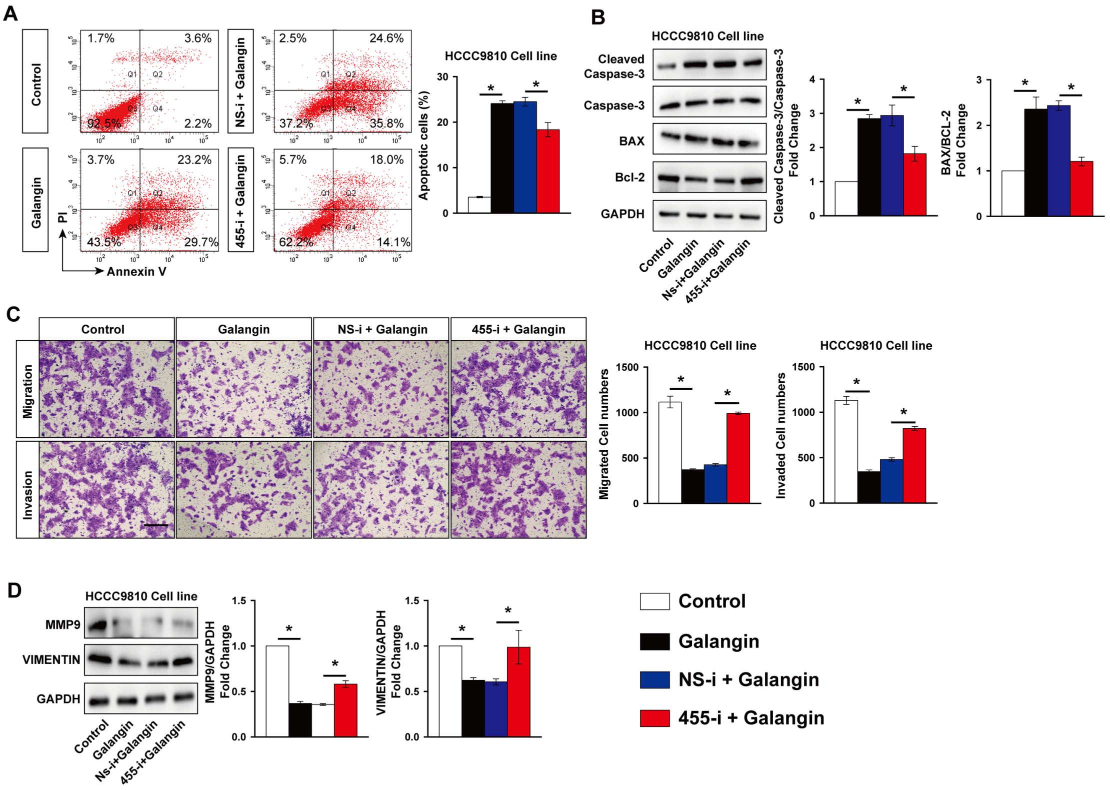

Supplemental Figure 7. Inhibition of miR-455-5p abrogates galangin's anti-cancer effects on CCA cell line HCCC9810 cells. HCCC9810 cells were transfected with miR-455-5p inhibitor (455-i) or non-specific inhibitor control (NS-i) at 100 nM for 24h followed by 150 μM galangin treatment for another 24h and harvested for (**A**) FACS analysis of apoptosis. (**B**) Western blot analysis of cleaved caspase 3, Caspase 3, Bax and Bcl-2 protein expression. (**C**) Matrigel-coated Transwell analysis of cell migration and invasion, scale bar, 50 μm. **(D)** Western blot analysis of MMP9 and Vimentin protein expression. **A to D**, n = 3 independent experiments. Values are given as means ± SEM. *$P < 0.05$.

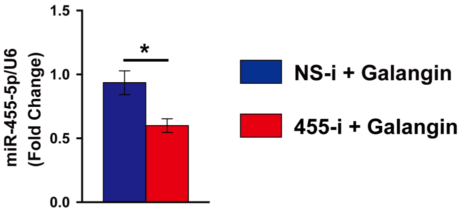

Supplemental Figure 8. The efficiency of miR-455-5p knockdown in xenograft model. Real-time PCR analysis of miR-455-5p expression in galangin treatment with 455-i or NS-i injection in the xenograft Balb/c nude mice model. n = 6 mice per group. Values were given as means ± SEM. *P < 0.05.
